# Lipoprotein succession in *Borrelia burgdorferi*: similar but distinct roles for OspC and VlsE at different stages of mammalian infection

**DOI:** 10.1111/mmi.12271

**Published:** 2013-06-07

**Authors:** Kit Tilly, Aaron Bestor, Patricia A Rosa

**Affiliations:** Laboratory of Zoonotic Pathogens, NIAID, NIH, Rocky Mountain LaboratoriesHamilton, MT, 59840, USA

## Abstract

*Borrelia burgdorferi* alternates between ticks and mammals, requiring variable gene expression and protein production to adapt to these diverse niches. These adaptations include shifting among the major outer surface lipoproteins OspA, OspC, and VlsE at different stages of the infectious cycle. We hypothesize that these proteins carry out a basic but essential function, and that OspC and VlsE fulfil this requirement during early and persistent stages of mammalian infection respectively. Previous work by other investigators suggested that several *B. burgdorferi* lipoproteins, including OspA and VlsE, could substitute for OspC at the initial stage of mouse infection, when OspC is transiently but absolutely required. In this study, we assessed whether *vlsE* and *ospA* could restore infectivity to an *ospC* mutant, and found that neither gene product effectively compensated for the absence of OspC during early infection. In contrast, we determined that OspC production was required by *B. burgdorferi* throughout SCID mouse infection if the *vlsE* gene were absent. Together, these results indicate that OspC can substitute for VlsE when antigenic variation is unnecessary, but that these two abundant lipoproteins are optimized for their related but specific roles during early and persistent mammalian infection by *B. burgdorferi*.

## Introduction

*Borrelia burgdorferi* cycles between mammalian and tick environments, each of which varies over time (see Radolf *et al*., 2012 for a recent review). When the spirochaetes are deposited in mammalian skin by a feeding tick, they must combat the host innate immune defences while replicating and spreading throughout the body of the mammal. As the mammal mounts an acquired immune response against *B. burgdorferi*, the spirochaete evades immune surveillance, at least in part, by changing components of its lipoprotein coat (Zhang *et al*., 1997; Crother *et al*., 2004; Liang *et al*., 2004). When a naive tick feeds on an infected mammal, the spirochaetes are acquired by the tick and establish infection in the midgut. As the tick digests the blood meal, the nutritional environment changes for the bacteria.

*Borrelia burgdorferi* genes important for nutrient acquisition and host adaptation are distributed throughout the complex *B. burgdorferi* genome, which is composed of a linear chromosome and multiple linear and circular plasmids (Fraser *et al*., 1997; Casjens *et al*., 2000; 2012). In addition to essential chromosomal genes, many genes encoding products important for growth in the mouse-tick infectious cycle are located on plasmids (Purser *et al*., 2003; Byram *et al*., 2004; Jewett *et al*., 2007b; 2009). Plasmid genes of unknown function are also important for the natural spirochaete life cycle (Labandeira-Rey *et al*., 2003; Grimm *et al*., 2004; Bankhead and Chaconas, 2007; Jewett *et al*., 2007a), but some entire plasmids are dispensable for bacterial growth in culture (Purser and Norris, 2000; Labandeira-Rey and Skare, 2001) and the loss of others may lead to small or negligible effects on infectivity in mice and ticks (Elias *et al*., 2002; Dulebohn *et al*., 2011).

Survival in the diverse mouse and tick environments requires adaptation by altering the gene expression and protein composition of the bacteria (Hodzic *et al*., 2002; 2003). Among the adaptations observed is a characteristic succession of outer surface lipoproteins (Schwan *et al*., 1995; Ohnishi *et al*., 2001; Liang *et al*., 2002b; Crother *et al*., 2004), whose genes are located on circular and linear plasmids (cp and lp respectively). Within infected ticks, the spirochaetes produce OspA (outer surface protein A) (Fingerle *et al*., 1995; Schwan *et al*., 1995), which contributes to maintaining tick colonization (Pal and Fikrig, 2003; Yang *et al*., 2004; Battisti *et al*., 2008) and is encoded on the 54 kb linear plasmid lp54. When nymphal ticks feed, the spirochaetes begin to synthesize OspC and reduce OspA synthesis (Schwan *et al*., 1995; Ohnishi *et al*., 2001). The *ospC* gene is located on the circular plasmid cp26 in the *B. burgdorferi* genome, and is required for the bacteria to initiate infection of a naive mammal (Grimm *et al*., 2004; Stewart *et al*., 2006; Tilly *et al*., 2006). OspC, however, is immunogenic and targeted by mammalian antibodies (Wilske *et al*., 1986; 1988), so bacteria that persist within an immunocompetent mammal must subsequently downregulate OspC (Liang *et al*., 2002a; 2004; Crother *et al*., 2004). Concomitant with the development of the host acquired immune response and OspC downregulation is increased synthesis of VlsE by *B. burgdorferi* (Hodzic *et al*., 2003; Crother *et al*., 2004). VlsE is a third lipoprotein, whose amino acid sequence does not resemble either OspC or OspA, but is abundantly present on the spirochaete surface during persistent infection (Crother *et al*., 2003). The *vlsE* locus is found on the 3′ end of the linear plasmid lp28-1, and has the key characteristic of undergoing antigenic variation by an error-prone gene conversion-like mechanism that utilizes the 15 silent *vlsE* cassettes found upstream of the *vlsE* locus on lp28-1 (Zhang *et al*., 1997; Zhang and Norris, 1998; Coutte *et al*., 2009). Because it is antigenically variable, VlsE protein can be present on the spirochaete surface during persistent infection of an immunocompetent host. Spirochaetes acquired by a larval tick feeding on a persistently infected small mammal reduce VlsE production in the tick midgut (Indest *et al*., 2001; Bykowski *et al*., 2006) and resume synthesis of OspA (Schwan *et al*., 1995; Hodzic *et al*., 2002).

Although this pattern of lipoprotein succession has been described, the functions of these outer surface proteins remain undefined. OspA plays a role in tick colonization by *B. burgdorferi* (Yang *et al*., 2004), and studies suggest that it is a tick midgut adhesin (Pal *et al*., 2000) and shields spirochaetes from mammalian antibodies in the incoming blood meal of feeding ticks (Battisti *et al*., 2008). OspC has been suggested to have roles in host selectivity (Brisson and Dykhuizen, 2004), plasminogen binding (Lagal *et al*., 2006; Onder *et al*., 2012), invasion (Lagal *et al*., 2006; Wormser *et al*., 2008), dissemination (Seemanapalli *et al*., 2010), salivary gland migration in the tick (Pal *et al*., 2004), evasion of innate immunity (Xu *et al*., 2008a), and recognition of the mammalian environment (Earnhart *et al*., 2010). Conflicting data exist as well for each of these proposed roles, and no well-supported function for OspC has emerged. *ospC* mutants have the clear phenotype of being defective at the initial phase of mammalian infection following needle inoculation or tick bite (Tilly *et al*., 2007; Dunham-Ems *et al*., 2012). An extensive study involving complementation of an *ospC* mutation with the genes for lipoproteins VlsE, OspA, and DbpA suggested that OspC provides a somewhat non-specific function, since substituting *ospC* with the other (unrelated) lipoprotein genes allowed spirochaetes to persist for several weeks after inoculation into SCID mice (Xu *et al*., 2008a). Further studies in immunocompetent mice suggested that the functions of these lipoproteins, while overlapping, may be optimized to the times at which they are naturally expressed, as constitutive expression of OspA and VlsE reduced infectivity of *B. burgdorferi* in mice (Xu *et al*., 2008b).

A simple model to explain these and other results is that these major *B. burgdorferi* outer surface lipoproteins serve a common function at different stages of the bacterial mouse-tick life cycle. In this model, that function is fulfilled by OspC during initial mammalian infection, by VlsE during persistent infection, and by OspA during the tick stage of the life cycle. The nature of that function may be protection against mammalian and tick innate immune defences. The present study describes experiments designed to further test the model that OspC and VlsE play similar roles at different stages of mammalian infection.

## Results

### Determining if OspC can substitute for VlsE

#### Mouse infection for assessing pC_p_*ospC* retention

The first set of experiments was designed to test whether OspC production must be sustained during infection when VlsE production is not possible. We pursued this question with an experimental design that was based on our previous studies with spirochaetes that contain lp28-1 and, therefore, produce VlsE (Tilly *et al*., 2006). In those studies, initial infection with an *ospC* mutant spirochaete required complementation with the *ospC* gene on a shuttle vector (which we call pC_p_*ospC*, to denote that it carries the *ospC* gene transcribed from the *ospC* promoter). The complementing plasmid was lost during persistent infection, indicating that *ospC* expression was not required at later times. In immunodeficient SCID mice, which lack B and T cells, the *ospC*-containing shuttle vector was more stable, most likely because these mice do not produce antibodies against OspC that could lead to clearance of spirochaetes that retained the plasmid and continued to express *ospC*. Nevertheless, significant shuttle vector loss was detected, indicating that sustained OspC production was not necessary during persistent infection of SCID mice either. In the present study, we reasoned that if VlsE normally fulfils the role of OspC during persistent infection, spirochaetes lacking VlsE would be unable to tolerate the absence of OspC during persistent infection. A measure of this intolerance would be strong positive selection for shuttle vector maintenance during persistent infection with a *B. burgdorferi* mutant lacking both the endogenous *vlsE* and *ospC* genes, but complemented with a shuttle vector carrying the *ospC* gene. Consequently, we screened for retention of the *ospC*-containing shuttle vector at a time at which we had previously found significant loss of the same shuttle vector by *ospC* mutant spirochaetes that retained lp28-1 and, therefore, *vlsE*. We predicted that spirochaetes lacking VlsE would retain the *ospC*-containing shuttle vector, in order to continue producing OspC in the absence of VlsE. Consistent with this hypothesis, a previous study with lp28-1-deficient spirochaetes found that these spirochaetes maintained OspC production during persistent infection of an immunodeficient host (Embers *et al*., 2008).

Wild-type (WT, C3H) and C3H-SCID mice were inoculated with 10^4^ spirochaetes per mouse of a strain lacking lp28-1 (*vlsE*^−^), a double mutant lacking both *vlsE* and *ospC* (*vlsE*^−^Δ*ospC*), and the same two strains containing the *ospC* shuttle vector pC_p_*ospC* (Table 1A). Six weeks post-inoculation, the mice were euthanized and spirochaete isolation from tissues was attempted. As expected, all strains were highly attenuated for persistent infection in WT mice (Table 3). Isolates were obtained from ankle joints from only two WT mice infected with *vlsE*^−^/pC_p_*ospC* and one WT mouse infected with *vlsE*^−^Δ*ospC*/pC_p_*ospC* (Table 3). However, all 10 WT mice inoculated with spirochaetes containing pC_p_*ospC* were seropositive (Table 3), indicating that they had been at least transiently infected. These data are consistent with previous studies showing that lp28-1 (carrying *vlsE*) is required for persistent infection of WT mice.

**Table 1A:** *Borrelia burgdorferi* strains used in this study

Strain	Description	Reference
WT (B31-A3)	Wild type, infectious B31 clone lacking cp9	Elias *et al*. (2002)
Δ*ospC (ospC*K1)	B31-A3 derivative with *ospC* deletion, kanamycin resistance cassette inserted in place of *ospC* locus (kan^r^)	Tilly *et al*. (2006)
B313	Non-infectious *B.* b*urgdorferi* strain lacking all linear plasmids except lp17	Sadziene *et al*. (1993)
B31-A34	Non-infectious B31 clone lacking lp5, lp25, lp28-1, 1p28-4, lp56, lp36, cp-9, and cp32-6	Jewett (2007a)
Δ*ospC*::C_p_*vlsE*	B31-A3 derivative with the *vlsE* gene coding sequence replacing the *ospC* ORF at the *ospC* locus on cp26 (kan^r^)	This study
*vlsE*^−^ (B31-A1)	Low passage B31 clone lacking lp28-1 (and *vlsE*)	Elias *et al*. (2002)
*vlsE*^−^Δ*ospC*	B31-A1 derivative with *ospC* deletion, kanamycin resistance cassette inserted in place of *ospC* locus (kan^r^)	This study

In contrast, spirochaetes lacking *vlsE* but containing *ospC* either at its normal location or on a shuttle vector (*vlsE*^−^, *vlsE*^−^/pC_p_*ospC*, and *vlsE*^−^Δ*ospC*/pC_p_*ospC*) persisted in all tissues in all SCID mice inoculated, whereas those lacking both *ospC* and *vlsE* (*vlsE*^−^Δ*ospC*) were not isolated from any SCID mouse tissues (Table 2). These findings were also consistent with previous studies, which demonstrated that lp28-1 was dispensable for persistent infection by *B. burgdorferi* in SCID mice (Lawrenz *et al*., 2004; Bankhead and Chaconas, 2007). Surprisingly, in this experiment, spirochaetes lacking just *ospC* (Δ*ospC*) were isolated from most SCID mice in which they were inoculated. This result differed from previous work and other experiments in this study, in which *ospC* was found to be an essential gene for normal infection of both immunocompetent and immunodeficient mice (Grimm *et al*., 2004).

#### Retention of pC_p_*ospC* in spirochaetes lacking *vlsE* during mouse infection

**Fig. 1:**
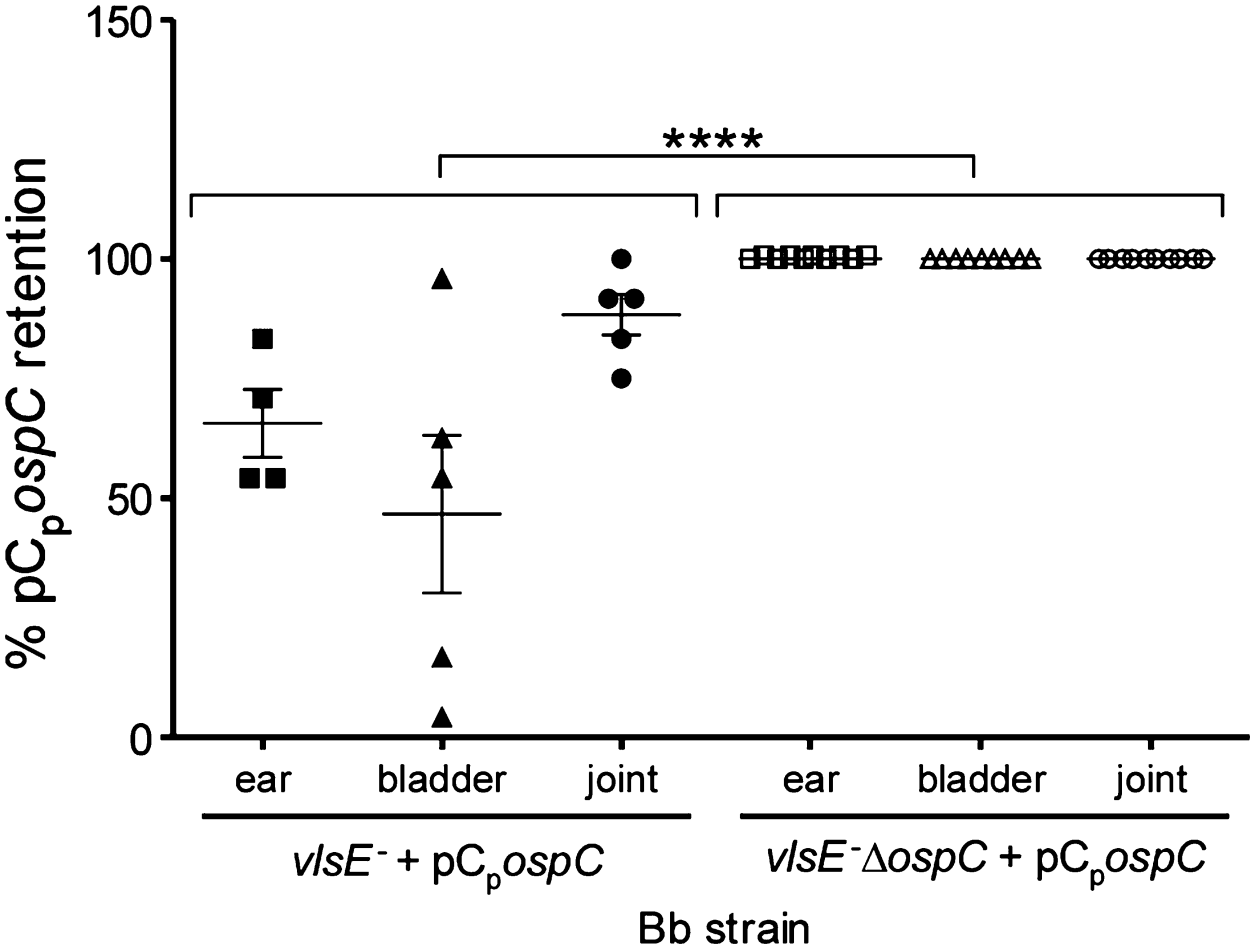
Shuttle vector retention in isolates from SCID mice. Percent retention of pC_p_*ospC* by *vlsE*^−^ vs *vlsE*^−^Δ*ospC* isolates from SCID mouse tissues 6 weeks post-inoculation. Each symbol denotes percent shuttle vector retention among 22–24 colonies screened per mouse isolate. All isolates of *vlsE*^−^Δ*ospC* retained pC_p_*ospC* in all colonies screened. The differences between the shuttle vector retention results for isolates of single *vlsE* mutant vs double *vlsE* and *ospC* mutant spirochaetes (*vlsE*^−^/pC_p_*ospC* vs *vlsE*^−^Δ*ospC*/pC_p_*ospC*) pooled from all tissues are statistically significant (*P* < 0.0001, as determined by the Mann–Whitney test). For one *vlsE*^−^Δ*ospC*/pC_p_*ospC* ear isolate, only 6 colonies were screened, all of which were shuttle vector-positive.

If sustained OspC production is required when VlsE production is not possible, spirochaetes lacking both *vlsE* and *ospC* but containing the *ospC*-carrying shuttle vector pC_p_*ospC* should retain that shuttle vector, even during infection of SCID mice. To ascertain if this were true, isolates obtained from mice infected with bacteria containing pC_p_*ospC* were plated for single colonies and colonies were screened by PCR for the presence of pC_p_*ospC*. In SCID mice, we obtained isolates from all tissues of all mice infected with *vlsE*-deficient or *vlsE*- and *ospC*-deficient spirochaetes containing pC_p_*ospC*, and compared shuttle vector retention in the presence and absence of the native *ospC* gene in bacteria lacking *vlsE*. We found significant loss of pC_p_*ospC* in SCID mice infected with spirochaetes lacking VlsE alone, but complete retention of the shuttle vector when the *vlsE*^−^ spirochaetes also lacked the endogenous *ospC* gene (Fig. 1) (*P* < 0.0001, as assessed by a two-tailed Mann–Whitney test or one-way anova). This result supports our hypothesis that continued OspC production would be required by bacteria lacking VlsE, if these two outer surface proteins fulfil the same essential function during infection of the mammalian host.

### Determining if VlsE or another outer-surface protein, OspA, can substitute for OspC

#### Generating *B. burgdorferi* with increased *vlsE* and *ospA* expression

**Fig. 2:**
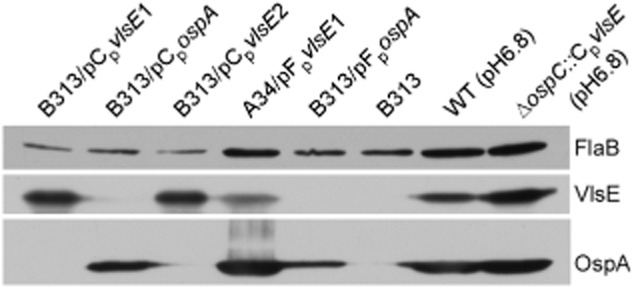
Immunoblot demonstrating VlsE and OspA production by spirochaetes carrying the shuttle vector constructs and the Δ*ospC*::C_p_*vlsE* strain. Antisera to VlsE, OspA and FlaB were used to probe *B. burgdorferi* lysates containing approximately 10^7^ spirochaetes. The pC_p_*vlsE*1, pC_p_*ospA*, pC_p_*vlsE*2, pF_p_*vlsE*1, and pF_p_*ospA* shuttle vectors were introduced into *B. burgdorferi* strains B313, which lacks native copies of *vlsE* and *ospA*, or B31-A34, which lacks a native copy of *vlsE*, to confirm production of these lipoproteins from their respective shuttle vector constructs. Wild type B31-A3 (WT), and Δ*ospC*::C_p_*vlsE*, which expresses *vlsE* from the *ospC* locus on cp26, were grown in pH 6.8 BSKII medium to late log phase to induce *vlsE* production from the *ospC* promoter.

Although OspA is neither required for nor typically produced during mammalian infection, studies by Xu *et al*. suggested that an *ospC* mutant could be partially complemented for mouse infectivity by several major outer-surface lipoproteins, including VlsE and OspA (Xu *et al*., 2008a,b). To confirm these results, we constructed several plasmids to determine if VlsE or another unrelated major outer-surface protein, OspA, could complement *ospC* mutant spirochaetes when establishing mammalian infection. Shuttle vectors pC_p_*vlsE*1 (*ospC* promoter-*vlsE*1) and pC_p_*ospA* (*ospC* promoter-*ospA*; Table 1B) encode *vlsE* and *ospA* expression from the *ospC* promoter. Shuttle vectors pF_p_*vlsE*1 (*flaB* promoter-*vlsE*1) and pF_p_*ospA* (*flaB* promoter-*ospA*) encode constitutive *vlsE* and *ospA* expression from the *flaB* promoter. Finally, shuttle vector pC_p_*vlsE*2 (*ospC* promoter-*vlsE*2) has the *ospC* promoter and signal sequence fused to the VlsE coding sequence (lacking its native signal sequence), to test if lipoprotein processing and membrane localization affects how well VlsE can fulfil the role of OspC (Table 1B). These shuttle vectors were introduced into *ospC* mutant *B. burgdorferi* (Δ*ospC*) to determine if OspA or VlsE, appropriately regulated or constitutively expressed, could fulfil the requirement for OspC in establishing mammalian infection. The shuttle vectors were also introduced into WT *B. burgdorferi* to investigate if inappropriate expression of these lipoproteins in the presence of native *ospC* expression would be deleterious for the spirochaetes. Finally, we verified that expression of the C_p_*vlsE* and C_p_*ospA* genes on the shuttle vector paralleled that of the endogenous *ospC* gene by introducing these constructs into the high-passage strain B313 (Table 1A) (Sadziene *et al*., 1993), which lacks native copies of the *ospA* and *vlsE* genes and constitutively produces OspC (Fig 2). We also confirmed the expression of OspA and VlsE from the *flaB* promoter fusion genes on the shuttle vector in *B. burgdorferi*, using strain B31-A34, which lacks the native *vlsE* gene (Jewett *et al*., 2007b) (Fig. 2). Although these experiments do not guarantee that the promoters on the shuttle vectors will behave identically to the endogenous *ospC* and *flaB* promoters when the spirochaetes infect a mammal, production of OspA and VlsE in cultured spirochaetes with these constructs correlated with what we know about expression of the endogenous promoters in the same culture conditions.

**Table 1B:** Shuttle vector constructs used in this study

Plasmid^a^	Bb promoter	Gene expressed	Reference
pC_p_*vlsE*1	*ospC*	*vlsE*	This study
pC_p_*vlsE*2	*ospC*	*ospC* signal sequence-*vlsE* fusion	This study
pF_p_*vlsE*1	*flaB*	*vlsE*	This study
pF_p_*ospA*	*flaB*	*ospA*	This study
pC_p_*ospA*	*ospC*	*ospA*	This study
pC_p_*ospC* (pBSV2G*ospC*)	*ospC*	*ospC*	Tilly *et al*. (2006)
pKFSS1	N/A^b^	N/A	Frank *et al*. (2003)

a pBSV2G, conferring gentamicin resistance, is the shuttle vector backbone used for all constructs, except for pKFSS1, which confers streptomycin resistance.

b N/A, not applicable.

#### OspA and VlsE do not fulfil the role of OspC in establishing mammalian infection in immunocompetent or immunodeficient SCID mice

Since the above results suggest that OspC is able to fulfil the role of VlsE (in the absence of an acquired immune response), we tested whether the converse were true, and VlsE could fulfil the role of OspC in establishing mammalian infection. We first used shuttle vectors carrying the VlsE or OspA open reading frames (ORFs) under the regulation of the *ospC* promoter. Groups of WT and C3H-SCID mice were intradermally injected with an inoculum of 10^4^ spirochaetes of WT or Δ*ospC* clones harbouring these shuttle vectors (Tables 1A and B). Immunocompetent mice were bled and all were euthanized three weeks later, and tissues were cultured for isolation of spirochaetes (Table 3). All WT mice that were positive by serology were also positive for *B. burgdorferi* isolation from all harvested tissues. Neither VlsE nor OspA was able to complement the Δ*ospC* mutant (Table 3). Overproduction of VlsE and OspA was inadequate for even transient infection, since uninfected animals were also seronegative. Furthermore, we found that inappropriate expression of *vlsE* (i.e. from an additional copy on the shuttle vector and under control of the *ospC* promoter) attenuated WT *B. burgdorferi* at a dose of 10^4^ in both WT and SCID mice (Table 3). This finding was similar to that of Xu *et al*., in which increased expression of *vlsE* led to clearance of the spirochaetes in immunocompetent mice (Xu *et al*., 2008b). To confirm that the reduced infectivity of spirochaetes carrying the *ospC* promoter-*vlsE* fusion on a shuttle vector was caused by inappropriate *vlsE* expression, we displaced the pC_p_*vlsE*1 shuttle vector with the incompatible plasmid pKFSS1 (Frank *et al*., 2003). The resultant WT/pKFSS1 strain exhibited WT infectivity (Table 3), indicating that the attenuated infectivity of WT/pC_p_*vlsE*1 was due to the *ospC* promoter-driven expression of *vlsE* from the shuttle vector.

Although neither VlsE nor OspA was capable of fulfilling the role of OspC when under control of the *ospC* promoter, the previous study of Xu *et al*. found that constitutive expression of these proteins in an *ospC* mutant background could restore infectivity to varying degrees in both WT and SCID mice (Xu *et al*., 2008a). Since our previous experiments utilized constructs with expression from the *ospC* promoter, we attempted to replicate the Xu *et al*. studies by introducing shuttle vector constructs with *vlsE* and *ospA* under control of the constitutive *flaB* promoter into both WT and Δ*ospC B. burgdorferi* (Table 4). VlsE production did not complement the Δ*ospC* mutant in either WT or SCID mice (Table 4), and uninfected animals were seronegative also, indicating that they had not been even transiently infected. OspA production was not able to complement the Δ*ospC* mutant in WT mice (0/5 mice infected and seronegative), as was observed by Xu *et al*. (2008a,b), but we did find a significant difference between the Δ*ospC* and the Δ*ospC/*pF_p_*ospA* strains in SCID mice (3/10 vs 5/5 mice infected respectively) (Table 4). However, in a separate experiment (Table 2), surprisingly, we found infection with the Δ*ospC* strain in 4 out of 5 SCID mice at a dose of 10^4^, so infection by Δ*ospC/*pF_p_*ospA* may not be a consequence of OspA production. Constitutive expression of *vlsE* did not attenuate WT *B. burgdorferi*, as we had seen with *ospC* promoter-driven expression of *vlsE* (Table 3). This may be the result of a lower expression level of VlsE from the pF_p_*vlsE*1 construct, since the *ospC* promoter is highly induced during early infection as compared with the *flaB* promoter (Tokarz *et al*., 2004).

**Table 2:** Infectivity of *B. burgdorferi* with various combinations of WT and mutant *ospC* and *vlsE* genes

*B. burgdorferi* strain	No. of persistently infected mice/no. of mice injected^a^	

	WT (serology)	SCID
*vlsE*^−^	0/5 (5/5)	10/10
*vlsE*^−^/pC_p_*ospC*^b^	2/5 (5/5)	5/5
Δ*ospC*	ND^c^	4/5
*vlsE*^−^Δ*ospC*	0/5 (0/5)	0/10
*vlsE*^−^Δ*ospC*/pC_p_*ospC*^b^	1/5 (5/5)	10/10

a Both immunocompetent (WT, C3H) and immunodeficient (C3H-SCID) mice were injected with 10^4^ spirochaetes of the indicated *B. burgdorferi* strain. Six weeks post-inoculation, WT mice were retro-orbitally bled and all mice were euthanized to assess infection by isolation of spirochaetes from ear, bladder, and ankle joints. Seroconversion of WT mice toward *B. burgdorferi* proteins is shown in parentheses, and indicates at least transient infection.

b Isolation of these strains from WT mice was only successful from ankle joints.

c ND, not determined, because the infection was not attempted in this experiment.

**Table 3:** Infectivity of *B. burgdorferi* expressing *vlsE* and *ospA* from the *ospC* promoter

*B. burgdorferi* strain	No. of infected mice/no. of mice injected^a^	

	WT	SCID
WT	6/8	9/10
WT/pC_p_*vlsE*1	2/10	0/5
WT/pC_p_*vlsE*2	1/5	ND^c^
WT/pC_p_*ospA*	4/5	5/5
WT/pKFSS1^b^	5/5	ND
Δ*ospC*	0/10	0/10
Δ*ospC*/pC_p_*vlsE*1	0/5	0/5
Δ*ospC*/pC_p_*vlsE*2	0/5	ND
Δ*ospC*/pC_p_*ospA*	0/5	0/5

a Both immunocompetent WT mice and immunodeficient C3H-SCID mice were injected intradermally with 10^4^ of the designated *B. burgdorferi* strain. At three weeks post-injection, WT mice were retro-orbitally bled to test for infection by immunoblot for seroconversion to *B. burgdorferi* proteins, and all mice were euthanized for attempted isolation of spirochaetes from the ear, bladder, and tibiotarsal joint. All WT mice positive by serology were also positive by isolation from all three tissues.

b The pBSV2G-*ospC*p-*vlsE* shuttle vector (pC_p_*vlsE*1) was displaced from the WT/pC_p_*vlsE*1 strain by introduction of the incompatible pKFSS1 shuttle vector. This strain was then injected into wild-type mice at a dose of 10^4^ to confirm that the loss of the pBSV2G-*ospC*p-*vlsE* shuttle vector restored infectivity to wild-type levels.

c No data. These strains were only tested in WT mice.

**Table 4:** Infectivity of *B. burgdorferi* expressing *vlsE* and *ospA* from the *flaB* promoter

*B. burgdorferi* strain	No. of infected mice/ no. of mice injected^a^	

	WT	SCID
WT	9/10	10/10
WT/pF_p_*vlsE*1	4/5	3/5
WT/pF_p_*ospA*	2/5	5/5
Δ*ospC*	0/10	3/10
Δ*ospC*/pF_p_*vlsE*1	0/5	0/5
Δ*ospC*/pF_p_*ospA*	0/5	5/5^b^

a Both immunocompetent WT mice and immunodeficient C3H-SCID mice were injected intradermally with 10^4^ organisms of the designated *B. burgdorferi* strain. At three weeks post-injection, WT mice were retro-orbitally bled to test by immunoblot for seroconversion to *B. burgdorferi* proteins, and all mice were euthanized for attempted isolation of spirochaetes from the ear, bladder, and tibiotarsal joint. All seropositive WT mice were also positive for isolation from all three tissues.

b The number of SCID mice infected by the Δ*ospC*/pF_p_*ospA* strain was significantly different from that of Δ*ospC* (*P* = 0.026, as determined by Fisher's two-tailed exact probability test).

#### Assaying the effect of replacing the endogenous *ospC* gene on cp26 with the *vlsE* gene expressed from the *ospC* promoter

In the experiments described above, complementation of an *ospC* mutant was attempted with genes located on shuttle vectors, which generally have slightly higher copy number than the endogenous genomic plasmids of *B. burgdorferi* (Tilly *et al*., 2006). To address the possibility that the lack of complementation (or attenuation of infection by WT *B. burgdorferi*) was a result of excess protein production due to higher copy number, we constructed a strain in which the *ospC* gene located on cp26 was replaced with the *vlsE* coding sequence, with the fusion between the *ospC* promoter at the start codon of the *vlsE* ORF (see *Experimental procedures*). Appropriate expression of the *vlsE* gene from the *ospC* promoter was assessed by growing spirochaetes *in vitro* at pH 6.8, in which conditions the WT *ospC* gene is typically induced. Although not entirely conclusive, a lysate of Δ*ospC*::C_p_*vlsE* (which has the normal *vlsE* gene, in addition to the *ospC*_p_*vlsE* fusion located on cp26) grown in these conditions appeared to contain more VlsE protein than was found in WT spirochaetes, as expected [Δ*ospC*::C_p_*vlsE* (pH 6.8), Fig. 2]. The lysate of WT bacteria grown in parallel contained more OspC than a lysate of uninduced WT *B. burgdorferi* (data not shown), confirming that the *ospC* promoter was induced in the conditions used. When tested for the ability to infect WT or SCID mice at an inoculum of ∼ 10^3^ per mouse, the Δ*ospC*::C_p_*vlsE* bacteria were non-infectious in both cases (Table 5). Not only were we unable to isolate the bacteria from any tissue, but the serology of the WT animals was negative (data not shown), indicating that the mice were not even transiently infected. In contrast, all WT and SCID mice were infected and colonized by WT *B. burgdorferi*. Again, we found that VlsE was unable to substitute for the absence of OspC, even when production of VlsE was directed by the *ospC* promoter at the normal locus on cp26 and, therefore, most likely to have been appropriate.

**Table 5:** Infectivity of *B. burgdorferi* with the *vlsE* ORF replacing the *ospC* ORF on cp26

*B. burgdorferi* strain	No. of persistently infected mice/no. of mice injected^a^	

	WT	SCID
WT	5/5	5/5
Δ*ospC*	1/5	0/5
Δ*ospC*::C_p_*vlsE*	0/5	0/5

a Both immunocompetent (IRW) and immunodeficient C3H-SCID mice were injected with ∼ 10^3^ organisms of the indicated *B. burgdorferi* strain. At three weeks post-inoculation, WT mice were retro-orbitally bled and tested for infection by immunoblot for seroconversion to *B. burgdorferi* proteins. All mice were euthanized and isolation of spirochaetes attempted from the ear, bladder, and ankle joint.

## Discussion

Although OspC has been shown to fulfil a critical role early in mammalian infection by *B. burgdorferi*, its function has remained undefined. This paper presents data addressing the model in which the function that OspC provides initially is required throughout infection by *B. burgdorferi*, and VlsE subsequently fulfils that function during persistent infection. We provide strong evidence that OspC production can compensate for VlsE deficiency in an immunodeficient host. We demonstrate that the *ospC* gene must be retained, and presumably expressed, when *vlsE* is missing. These data imply that OspC can carry out the function of VlsE during persistent infection, provided it is not targeted by acquired immunity. However, although OspC is able to substitute for VlsE in an immunodeficient host, we found that VlsE could not substitute for OspC in any host background. Therefore, our data indicate that the two proteins are not strictly interchangeable, so the simplest model of redundant function with reciprocal expression is not correct.

Although this study does not support the idea that VlsE can also substitute for OspC, some data do suggest that other *B. burgdorferi* proteins can take the place of OspC during initiation of mammalian infection. One of the strongest pieces of evidence is apparently normal infection of mice by an *ospC* deletion mutant when tissue from a persistently infected animal was transferred to a naive animal (Tilly *et al*., 2006). We previously speculated that VlsE, which is made by the host-adapted spirochaetes, allows infection of the naive animal in this scenario. Xu *et al*. (2008a) partially restored infectivity of an *ospC* mutant by complementation with VlsE, and also suggested that the proteins have similar functions. However, we were not able to duplicate their findings (A. Bestor, unpubl. results), even when using the same *vlsE* allele as Xu *et al*.

We also found that *ospA* was unable to fully complement an *ospC* mutation. Although we found apparent complementation of the *ospC* mutation by *ospA* driven by the *flaB* promoter in SCID mice, the ability of Δ*ospC* spirochaetes to infect SCID mice on occasion (see, e.g. Table 2and below) means that this result may have been spurious. Clearly, there are differences between our system and that of Xu *et al*., who did find complementation of an *ospC* mutation by overexpression of the *vlsE* and *ospA* genes (Xu *et al*., 2008a). For example, the typical ID50 of *in vitro*-grown WT *B. burgdorferi* is almost 100-fold higher in our experiments than in theirs. Also, the *ospC* mutant used by Xu *et al*. (2008a) is an insertion, rather than a deletion of the entire coding sequence, and it lacks lp25 but carries the essential gene *pncA* on the *ospC*-complementing plasmid, which requires that the shuttle vector be retained during mammalian infection. Nevertheless, in our experiments, VlsE and OspA did not fulfil the role of OspC for initiation of infection.

It remains unclear why OspC can substitute for VlsE but not vice versa. OspC may perform a unique role early in infection, in addition to a common function that VlsE typically assumes as the spirochaetes persist in a mammal. Without invoking an additional unknown role, VlsE could be unable to take the place of OspC because the lipoprotein requirement during initial infection by *B. burgdorferi* is more stringent, since few bacteria are present and they are all located in the tick bite (or needle inoculation) site.

Because it appears that OspC and VlsE are not fully interchangeable, we propose that these two proteins require both appropriate context and appropriate timing to be fully functional. By context, we encompass the roles of other proteins produced at the same time and also variations in membrane composition and arrangement, which are influenced by lipid availability and temperature. Although the OspC and VlsE sequences are not related, the tertiary structures of the OspC dimer and VlsE monomer are similar in size and shape, with variable regions forming surface-exposed loops (Eicken *et al*., 2001; 2002; Kumaran *et al*., 2001), which may allow them to carry out a common function. As to the question of what their common function could be, OspC and VlsE might serve to protect the bacterium from the particular host environment in which it finds itself, or they might stabilize the bacterial structure, again in the particular host context. If OspC and VlsE protect *B. burgdorferi* against host defences, which defences those might be remains undefined. We have looked without success for differences between *ospC* mutant and WT spirochaetes with respect to phagocytosis by mouse and human neutrophils, phagocytosis by mouse macrophages, susceptibility to human or mouse complement, or susceptibility to mouse natural killer cells (K. Tilly, A. Porter, F. DeLeo, C. Checroun, and P. Rosa, unpubl. data). Although these studies were not exhaustive, they emphasized the complexity of the interaction between host and bacterium and the inherent limitations of investigating that interaction either with isolated components or *in vivo*. If OspC and VlsE stabilize the bacteria in the host environment, large amounts of a particular lipoprotein may be an essential component of the recently described *B. burgdorferi* lipid rafts (LaRocca *et al*., 2010). Given that the *B. burgdorferi* membrane structure and composition vary depending on the host environment, it would not be surprising if an essential lipoprotein component would also need to vary.

Although we did not succeed in heterologous complementation of the *ospC* deletion mutation, we did occasionally observe infection of SCID mice by Δ*ospC* spirochaetes. Furthermore, in recent experiments we have sometimes obtained infection in both WT and SCID mice with Δ*ospC* spirochaetes when inoculating at a dose ≥ 10^7^ spirochaetes, confirmed by isolation of the mutant from all cultured tissues (A. Bestor, K. Tilly and P. Rosa, unpubl. results). This is in contrast to the complete lack of infectivity by *ospC* mutant spirochaetes that we found in earlier studies (Grimm *et al*., 2004; Stewart *et al*., 2006; Tilly *et al*., 2006). *ospC* mutant spirochaetes grown directly from stocks frozen since the strain was originally isolated exhibit a similar phenotype (K. Tilly, unpubl. results), so we do not think that the Δ*ospC* mutant used in this study has undergone a compensatory mutation. Since the earlier tissue transfer studies suggest that a *B. burgdorferi* protein produced during persistent infection, when appropriately regulated and in the correct context, can successfully fulfil the *ospC* requirement (Tilly *et al*., 2009), our culture conditions may have shifted subtly to increase production of that product and its appropriate context by Δ*ospC* spirochaetes. We have tested several batches of medium, and growth to different densities, but have not pinpointed a tangible variable that affects infectivity of the *ospC* mutant. Despite occasional infection by the *ospC* mutant at high dose, we have been unable to demonstrate consistently increased infectivity at a standard inoculum when the mutant was complemented with either *vlsE* or *ospA*.

This and other studies are delineating a succession of lipoproteins that coat *B. burgdorferi* and provide essential functions in the mouse and tick hosts at various stages of the bacterial life cycle. OspC is typically on the bacterial surface when spirochaetes initiate mammalian infection, and then subsequently downregulated. VlsE is produced and undergoes antigenic variation during persistent infection. OspA is required during bacterial colonization of ticks. Each has unique expression and protein characteristics, yet their roles may be related. The bacteria are exposed to different degrees of immune surveillance throughout the infectious cycle, from dermal inoculation of the mammal, to blood stream dissemination and peripheral tissue colonization, and acquisition with the blood of a seropositive host by the feeding tick. The spirochaetes also will have different protein and lipid composition at these stages, which could affect bacterial stability and survival in the host environment. The changing spirochaete surface, of which the major lipoproteins are essential components, could be considered to be analogous to the more complex developmental changes found in vector-borne parasites during their mammalian and arthropod phases. Further defining the shared or specific roles of major outer surface lipoproteins will help elucidate the details of the *B. burgdorferi* infection process and, in so doing, reveal potentially widespread adaptations to the host and vector environments.

## Experimental procedures

### Bacterial strains and culture conditions

*Borrelia burgdorferi* strains were derived from clones B31-A3 or B31-A1 (Elias *et al*., 2002), which we refer to as WT and *vlsE*^−^ respectively. Both B31-A3 and B31-A1 are clones derived from non-clonal B31 MI (Fraser *et al*., 1997), but B31-A3 lacks cp9, whereas B31-A1 lacks both cp9 and lp28-1 (see Table 1A for strain descriptions). Strain *ospC*K1 (referred to as Δ*ospC*) (Tilly *et al*., 2006) is a derivative of WT B31-A3 in which the *ospC* gene is deleted and replaced with a *flgB*p-*kan* fusion. *B. burgdorferi* liquid cultures were grown in BSKII medium at 35°C. Electrotransformation was performed as described (Samuels, 1995), using 5–10 μg DNA. Selection for transformants was in solid BSK medium containing 200 μg ml^−1^ kanamycin, 50 μg ml^−1^ streptomycin, or 40 μg ml^−1^ gentamicin. DNA manipulations in *Escherichia coli* were performed with TOP10 cells (Invitrogen, Carlsbad, CA) or NEB5α (New England Biolabs, Ipswich, MA).

To make strains for testing whether OspC could fulfil the role of VlsE, the *ospC* gene in *vlsE^−^*, which lacks lp28-1 and the encoded *vlsE* locus (Table 1A), was inactivated by allelic exchange using plasmid pJK109 (Tilly *et al*., 2006). This plasmid includes *ospC* flanking sequences surrounding *flgB*_p_-*kan*, which replaces the *ospC* gene. We chose a transformant (*vlsE^−^*Δ*ospC*) that had the same plasmid content as *vlsE^−^*, but lacked the *ospC* ORF. *vlsE^−^* and *vlsE^−^*Δ*ospC* were transformed by electroporating with pBSV2G*ospC* (referred to as pC_p_*ospC*), a shuttle vector carrying the *ospC* gene and its own promoter (Table 1B) that had been methylated (Rego *et al*., 2011), and selecting for gentamicin-resistance. Transformant clones with the same plasmid content as *vlsE^−^* and containing pC_p_*ospC* were used for subsequent experiments.

### Construction of shuttle vectors carrying lipoprotein genes fused to various promoters

The pC_p_*vlsE*1 shuttle vector, expressing *vlsE* under control of the *ospC* promoter, was constructed as follows. The *ospC* promoter was amplified from *ospC*7 (Grimm *et al*., 2004) genomic (g) DNA with Vent polymerase (New England Biolabs) using primers 1 and 2 (Table 6) and ligated into the KpnI and XbaI sites in the multiple cloning site (MCS) of pBSV2G (Elias *et al*., 2003), yielding pBSV2G-*ospC*p. The region of lp28-1 encompassing the *vlsE* ORF was amplified with *Taq* polymerase, using primers 3 and 4 (Table 6), from WT gDNA that had been treated with Mung Bean nuclease (New England Biolabs) to nick hairpin ends, and ligated into BspHI-digested pBSV2G-*ospC*p, yielding pBSV2G-*ospC*p-*vlsE* (pC_p_*vlsE*1).

**Table 6:** Primers used in this study

Primer	Designation	Sequence^a^
1	*ospC*p 5′ KpnI	**GGTACC**GGGGTACCAAGTATTGCCTGAGTATTC
2	*ospC*p 3′ BspHI	GCGCGC**TCATGA**ATTTGTGCCTCCTTTTTAT
3	*vlsE* 5′ BspHI	CGCGCG**TCATGA**AAAAAATTTCAAGTGC
4	*vlsE* 3′ XbaI	GC**TCTAGA**TCTCGACTATTTCCTCAATC
5	*ospC*p(+ss) 3′ XbaI	**TCTAGA**CCCTGAATTATTACAACGATATAAATAAAAA
6	*vlsE* 3′ HindIII	**AAGCTT**TCACTTATTCAAGGCAGGAG
7	*vlsE*(−ss) 5′ XbaI	**TCTAGA**AAAAGCCAAGTTGCTGATAA
8	*flaB*p 5′ XbaI	**TCTAGA**TTATTTGCCGACTACCTTGG
9	*flaB*p 3′ XbaI/BspHI	**TCTAGA**GG**TCATGA**ATATCATTCCTCCATGATAAA
10	*vlsE* 3′ SphI	**GCATGC**TCACTTATTCAAGGCAGGAGGTGTTTCTTTACTAGCAGCC
11	*ospA* 5′ BspHI	**TCATGA**AAAAATATTTATTGGGAATAGGTCTAATATTAGCCT
12	*ospA* 3′ SphI	GGG**GCATGC**TTATTTTAAAGCGTTTTTAATTTCATCAAGTTTGTAATT
13	*vlsE* 3′ NotI	**GCGGCCGC**TCACTTATTCAAGGCAGGAGGTGTTTCTTT
14	*flg*5′-AvrII	**CCTAGG**TAATACCCGAGCTTCAAGGAG
15	*aacC1*3′-NheI	**GCTAGC**CGATCTCGGCTTGAACG

a Restriction enzyme recognition sequences are indicated in bold type.

The pC_p_*vlsE*2 shuttle vector, expressing and transporting VlsE under the control of the *ospC* promoter and carrying the *ospC* signal sequence, was constructed as follows. The region of cp26 from nucleotides 16647–16971, encompassing the *ospC* promoter and *ospC* signal sequence, was amplified from WT gDNA with Vent polymerase using primers 1 and 5 (Table 6), and ligated into the KpnI and XbaI sites found in the MCS of pBSV2G, yielding pBSV2G-*ospC*p(+ss). The *vlsE* locus was amplified without the predicted signal sequence from pC_p_*vlsE*1 with Vent polymerase using primers 6 and 7 (Table 6), and ligated into XbaI/HindIII-digested pBSV2G-*ospC*p(+ss), yielding pBSV2G-*ospC*p(+ss)-*vlsE* (pC_p_*vlsE*2).

The pF_p_*vlsE*1 shuttle vector, constitutively expressing *vlsE* from the *flaB* promoter, was constructed as follows. The *flaB* promoter was amplified from WT gDNA with Vent polymerase using primers 8 and 9 (Table 6), and ligated into the XbaI site in the MCS of pBSV2G, yielding pBSV2G-*flaB*p. The region of lp28-1 encompassing the *vlsE* locus was amplified from pC_p_*vlsE*1 with Vent polymerase using primers 3 and 10 (Table 6), and ligated into BspHI/SphI-digested pBSV2G-*flaB*p, yielding pBSV2G-*flaB*p-*vlsE* (pF_p_*vlsE*1).

The pC_p_*ospA* shuttle vector, expressing *ospA* under control of the *ospC* promoter, was constructed as follows. The region of lp54 from nucleotides 9457 to 10278, encompassing the *ospA* ORF, was amplified from WT gDNA with Vent polymerase using primers 11 and 12 (Table 6), and ligated into BspHI/SphI-digested pBSV2G-*ospC*p, yielding pBSV2G-*ospC*p-*ospA* (pC_p_*ospA*).

The pF_p_*ospA* shuttle vector, constitutively expressing *ospA* from the *flaB* promoter, was constructed as follows. The region of lp54 from nucleotides 9457 to 10278, encompassing the *ospA* locus, was amplified from WT gDNA with Vent polymerase using primers 11 and 12 (Table 6), and ligated into BspHI/SphI-digested pBSV2G-*flaB*p, yielding pBSV2G-*flaB*p-*ospA* (pF_p_*ospA*).

All constructs were sequenced upon completion to confirm they were as designed and free of any mutations before transforming them into the WT and the Δ*ospC* strains (see Table 1), and selecting with gentamicin. *B. burgdorferi* plasmid contents of the transformants were confirmed to be the same as the parental strains by PCR (Akins *et al*., 1998).

### Plasmid and strain construction to replace the *ospC* ORF on cp26 with the *vlsE* ORF

To make a plasmid for replacing the *ospC* ORF with the *vlsE* ORF at the *ospC* locus on cp26, we began with pJK109, a plasmid previously used to inactivate *ospC* by allelic exchange (Tilly *et al*., 2006). The *ospC* upstream fragment was modified at the 3′ end by addition of a *Bsp*HI site that included the ATG start codon of OspC, and was generated by PCR using primers 2 (Table 6) and JK170 (Tilly *et al*., 2006). We amplified the *vlsE* gene from the pC_p_*vlsE*1 plasmid using primers 3 and 13 (Table 6). Both of these fragments were cloned into PCR2.1Topo. The amplified fragments were digested from the vector using *Sac*I-*Bsp*HI and *Bsp*HI-*Not*I, respectively, and ligated (in a three-way ligation) with *Sac*I-*Not*I digested JK109. A plasmid with the correct fusion was named pOVK.

The *ospC* promoter-*vlsE* ORF fusion was moved into *B. burgdorferi* by transforming WT bacteria with approximately 5 μg of methylated pOVK (Rego *et al*., 2011). Three colonies were obtained, of which one had the appropriate allelic replacement at the *ospC* locus on cp26. When the plasmid content of this strain was checked, it was found to have lost lp25. Accordingly, the clone was transformed with 10 μg of total genomic DNA from A3 lp25-Sm, which has a *flgB*_p_-*aadA* fusion (Frank *et al*., 2003) inserted into lp25 at the site used in A3 lp25-Gm (Grimm *et al*., 2005). A strep^R^ colony in which lp25 had been restored was picked and used for subsequent experiments.

### Mouse infections

All animal experiments were performed using protocols approved by the Animal Care and Use Committee of the Rocky Mountain Laboratories and according to the guidelines of the National Institutes of Health. Rocky Mountain Laboratories is accredited by the International Association for Assessment and Accreditation of Laboratory Animal Care (AAALAC). C3H/HeN and C3H/HeN SCID mice were purchased from Harlan Sprague-Dawley (Indianapolis, IN). RML mice are outbred, derived from Swiss-Webster mice, and bred at the Rocky Mountain Laboratories. IRW mice are inbred, derived from RML mice, and also bred at the Rocky Mountain Laboratories. For infection studies, mice were injected with 80% of the inoculum intraperitoneally and 20% of the inoculum subcutaneously. In some cases (as indicated in the Results), bacteria were inoculated into the intradermal-subcutaneous compartment, with the appropriate number of bacteria provided in a single 100 μl injection with a 27 ga needle. The mice were euthanized three to six weeks post-inoculation, and ears, bladders, and ankle joints harvested and cultured in 10 ml BSKII medium. Isolates from mice infected with *vlsE^−^*/pC_p_*ospC* or *vlsE^−^*Δ*ospC*/pC_p_*ospC* were plated in solid medium and resultant colonies were screened by PCR with primers 14 and 15 (Table 6), to assess retention of pC_p_*ospC* (Tilly *et al*., 2009).

### SDS PAGE and immunoblot analysis

*Borrelia burgdorferi* protein lysates (approximately 10^7^ spirochaete-equivalents per well) were separated in 12.5% SDS-polyacrylamide gels and immunoblotted as described (Bestor *et al*., 2012). Detection was with SuperSignal West Pico chemiluminescent substrate (Thermo Sicentific, Rockford, IL). Antibodies were as follows: mouse monoclonal anti-FlaB H9724 (Barbour *et al*., 1986), rabbit polyclonal anti-VlsE (Bykowski *et al*., 2006), and mouse monoclonal anti-OspA H5332 (Barbour *et al*., 1983). Sera from inoculated animals were diluted 1:200 for assessing seroreactivity.
